# Nb_2_O_5_-γ-Al_2_O_3_ nanofibers as heterogeneous catalysts for efficient conversion of glucose to 5-hydroxymethylfurfural

**DOI:** 10.1038/srep34068

**Published:** 2016-09-26

**Authors:** Huanfeng Jiao, Xiaoliang Zhao, Chunxiao Lv, Yijun Wang, Dongjiang Yang, Zhenhuan Li, Xiangdong Yao

**Affiliations:** 1 Collaborative Innovation Center for Marine Biomass Fibers, Materials and Textiles of Shandong Province; College of Chemistry, Chemical and Environmental Engineering, Qingdao University, Qingdao 266071, China; 2 Queensland Micro- and Nanotechnology Centre (QMNC), Griffith University, Nathan, Brisbane, QLD 4111, Australia; 3 State Key Laboratory of Separation Membranes and Membrane Processes, School of Materials Science and Engineering, Tianjin Polytechnic University, 300160 Tianjin, China

## Abstract

One-dimensional γ-Al_2_O_3_ nanofibers were modified with Nb_2_O_5_ to be used as an efficient heterogeneous catalyst to catalyze biomass into 5-hydroxymethylfurfural (5-HMF). At low Nb_2_O_5_ loading, the niobia species were well dispersed on γ-Al_2_O_3_ nanofiber through Nb–O–Al bridge bonds. The interaction between Nb_2_O_5_ precursor and γ-Al_2_O_3_ nanofiber results in the niobia species with strong Lewis acid sites and intensive Brønsted acid sites, which made 5-HMF yield from glucose to reach the maximum 55.9~59.0% over Nb_2_O_5_-γ-Al_2_O_3_ nanofiber with a loading of 0.5~1 wt% Nb_2_O_5_ at 150 °C for 4 h in dimethyl sulfoxide. However, increasing Nb_2_O_5_ loading could lead to the formation of two-dimensional polymerized niobia species, three-dimensional polymerized niobia species and crystallization, which significantly influenced the distribution and quantity of the Lewis acid sites and Brönst acid sites over Nb_2_O_5_-γ-Al_2_O_3_ nanofiber. Lewis acid site Nb^δ+^ played a key role on the isomerization of glucose to fructose, while Brønsted acid sites are more active for the dehydration of generated fructose to 5-HMF. In addition, the heterogeneous Nb_2_O_5_-γ-Al_2_O_3_ nanofiber catalyst with suitable ratio of Lewis acid to Brönsted sites should display an more excellent catalytic performance in the conversion of glucose to 5-HMF.

Fossil-based resources such as petroleum, coal and natural gas are deemed as the dominant raw materials to be used for energy and synthesis of organic chemicals^1^,^2^. Nevertheless, the mismatch between the increasing demand for and sharply diminishing supply of fossil-based resources implies that the search for alternative raw material sources is critically important. Renewable biomass is the most suitable candidate for alternative raw material sources since they are abundant, easy to obtain and rich of carbohydrates which can be converted to valuable chemicals^3^,^4^. Consequently, the conversion of renewable biomass to fuels and chemicals has received wide attention.

Cellulose as an important branch of biomass is composed of the basic glucose unit building blocks that can be transformed to the useful platform molecule 5-HMF. 5-HMF can acts as the raw material to be used to synthesize chemicals, liquid fuels and so on ref. 5 and 6. Hence, developing an approach to efficiently synthesize 5-HMF from rich and cheap glucose resources under mild conditions is extremely desirable.

5-HMF synthesis from glucose is difficult due to the high stability of the glucose ring, making the dehydration process more difficult^7^. In order to overcome this disadvantage, the catalysts were paid more attention to reduce the activation energy of this reaction. Catalysts used for glucose dehydration to 5-HMF are classified into heterogeneous and homogeneous catalysts which play the different performance in different reaction solvents such as aqueous, organic solvents and ionic liquids^8^,^9^. Homogeneous catalysts like ionic liquids and metal salts employed for 5-HMF conversion from glucose are limited due to several drawbacks including the high cost, toxicity, difficult separation and recovery^10^,^11^. In contrast, heterogeneous catalysts avoided aforementioned disadvantage have been widely utilized for biomass conversion into 5-HMF, e.g. oxides, phosphates, ion exchange resins and heteropolyacids^12^,^13^.

As for the heterogeneous catalysts, besides the main catalytic active sites like metal oxides such as WO_3_, TiO_2_, and ZrO_2_, the supports also play a very important role on catalytic process^14^,^15^. Generally, the conventional porous materials were used as supports because of their large surface areas. But the porosity and surface area will be reduced during the loading of active sites. Recently, various one-dimensional (1D) oxide nanofibers have been reported as the efficient heterogeneous catalyst supports, which can realize the loading of active sites without declining surface area^16^,^17^.

Herein, the active sites (acidic Nb_2_O_5_) were loaded on the surface of 1D γ-Al_2_O_3_ nanofibers by facile incipient-wetness impregnation method. Nb_2_O_5_-γ-Al_2_O_3_ nanofibers displayed the high catalytic activity in glucose conversion to 5-HMF with dimethyl sulfoxide as solvent, and it was found that Lewis acid site Nb^δ+^ promoted the isomerization of glucose to fructose, while Brønsted acid sites catalyzed the dehydration of generated fructose to 5-HMF.

## Results and Discussion

### Nb_2_O_5_/γ-Al_2_O_3_ nanofiber characterization

1D γ-Al_2_O_3_ nanofiber with different Nb_2_O_5_ loading of 0, 1, 3, 3.4, 4.7, 9.4, 26.6 and 33.9 wt% was prepared by facile incipient-wetness impregnation method. Table 1 presents the contents of Nb_2_O_5_ measured by ICP-AES technique (after the samples were dissolved by strong phosphoric acid). The characterized results reveal that the actual Nb_2_O_5_ contents are same or smaller than the controlled Nb_2_O_5_ content.

**Figure 1:**
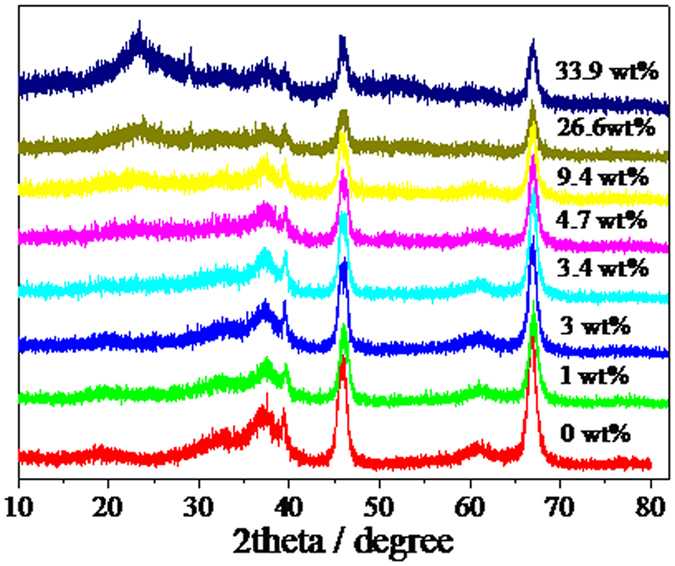
XRD of γ-Al_2_O_3_ with different Nb2O_5_ loadings.

XRD patterns were collected from 5 to 80° (Fig. 1). It is found that the distinct diffraction peaks of these samples appear at 37.6°, 39.5°, 45.8°, 60.9° and 67°, which are assigned to the diffraction of (3 1 1), (2 2 2), (4 0 0), (5 1 1) and (4 4 0) of γ-Al_2_O_3_, respectively. With the increase of Nb_2_O_5_ loading, the intensive diffraction peaks at 2θ = 22.6° and 28.5° appeared, which are assigned to the diffraction of bulk Nb_2_O_5_^18^.

**Figure 2:**
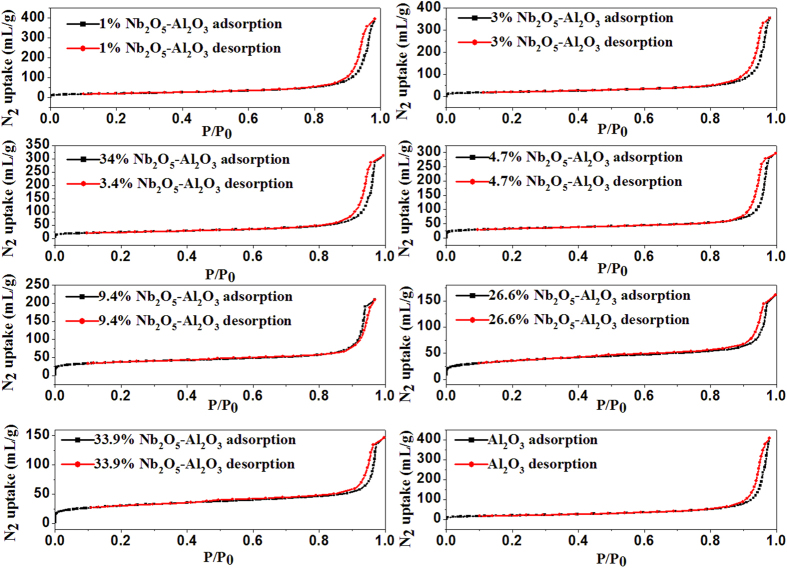
The N_2_ sorption isotherms at 77 K for γ-Al_2_O_3_ (solid circles: adsorption; open circles: desorption).

Textural properties of catalysts obtained from the nitrogen sorption at 77 K are listed in Table 2 and Fig. 2. These isotherms are similar with each other which belong to the type IV Van Der Waals isotherm^19^. Both pore volume and average pore diameters decrease with the increase of Nb_2_O_5_ loading, but the BET surface areas of Nb_2_O_5_-γ-Al_2_O_3_ nanofibers gradually increase with Nb_2_O_5_ loading augment. However, further increasing Nb_2_O_5_ loading will lead to BET surface area decrease, which is similar with the report of literature^20^.

**Figure 3:**
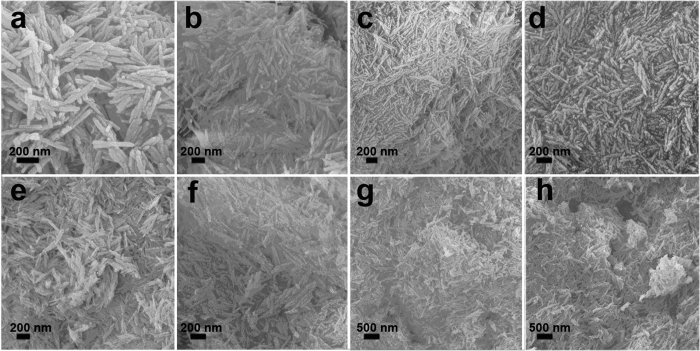
SEM images of γ-Al_2_O_3_ with different Nb_2_O_5_ loadings: (**a**) 0 wt%; (**b**) 1 wt%; (**c**) 3 wt%; (**d**) 3.4 wt%; (**e**) 4.7 wt%; (**f**) 9.4 wt%; (**g**) 26.6 wt%; (**h**) 33.9 wt%.

**Figure 4:**
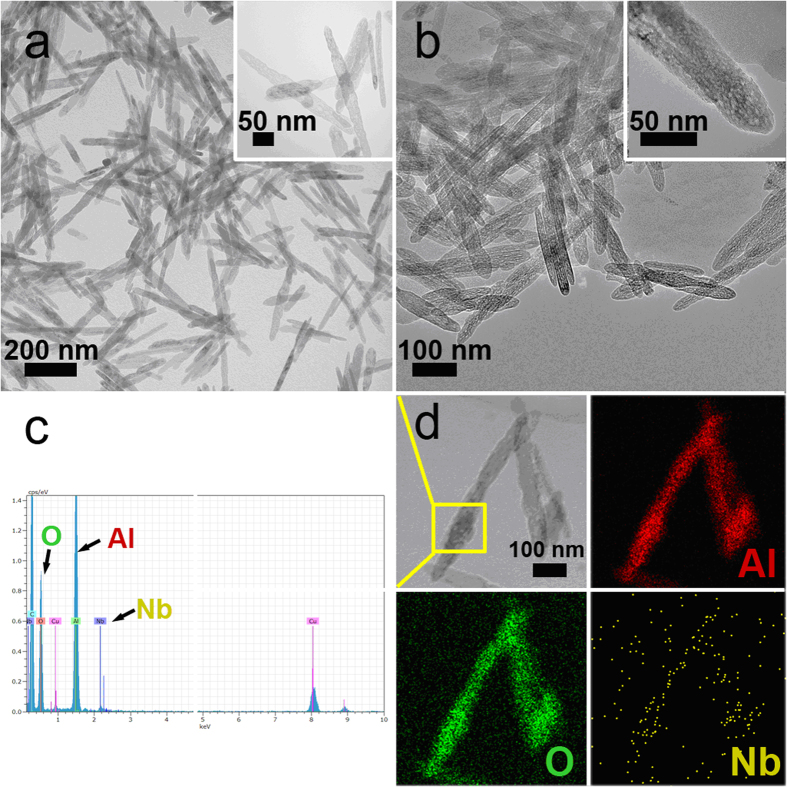
TEM images of γ-Al_2_O_3_ with different Nb_2_O_5_ loadings: (**a**) 0 wt%; (**b**) 1 wt%. (**c**) EDX patterns of the selected area of the 1 wt% Nb_2_O_5_-γ-Al_2_O_3_. (**d**) The TEM image and elemental mapping of the 1 wt% Nb_2_O_5_-γ-Al_2_O_3_.

The scan electron microscopy (SEM) images of γ-Al_2_O_3_ nanofiber with different Nb_2_O_5_ loading from 0 to 33.9 wt% are presented in Fig. 3a–h. All samples have the homogeneous length of 200–300 nm and the similar diameter of 30–50 nm, which indicates Nb_2_O_5_ loading do not influence the morphology and structure of γ-Al_2_O_3_ nanofibers. However, the particles are observed to aggregate together when Nb_2_O_5_ loading reaches 4.7 wt%, due to the surface tension^21^,^22^. Moreover, the characterization of γ-Al_2_O_3_ and 1 wt% Nb_2_O_5_-γ-Al_2_O_3_ by transmission electron microscopy (TEM) was also performed. As shown in Fig. 4a,b, the average size and the morphology of these particles are similar. Elemental mapping by EDS was used to study the distribution of the Al, O, and Nb elements in nanofiber based samples (Fig. 4d). The abundant Al and O elements distribute homogeneously in a single nanofiber, however the content of Nb element is comparably smaller and Nb element mainly distributes on the surface of γ-Al_2_O_3_.

**Figure 5:**
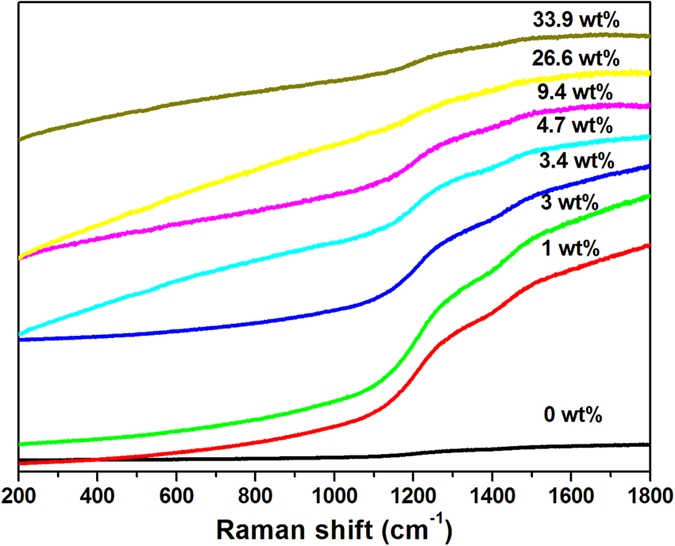
Raman spectra of γ-Al_2_O_3_ with different Nb_2_O_5_ loadings.

As shown in Fig. 5, γ-Al_2_O_3_ nanofiber exhibits the very weak Raman bands in the region of 200~2000 cm^−1^ due to the low polarizability of light atoms and the ionic character of Al–O bonds^23^. As for Nb_2_O_5_-γ-Al_2_O_3_ nanofiber with 1~9.4 wt % load, the intensive Raman bands in the region of 1000~2000 cm^−1^ appeared due to the polarizability of Nb–O–Al species^24^.

### Effects of different Nb_2_O_5_ loading on biomass selectivity conversion

**Figure 6:**
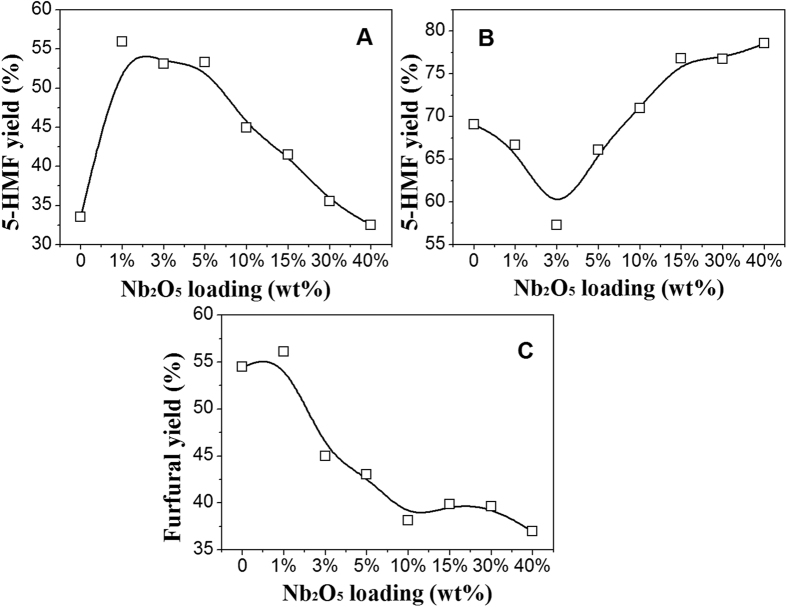
Effect of different Nb_2_O_5_ loadings on γ-Al_2_O_3_ on the yield of: 5-HMF from the dehydration of glucose at for 4 hours (**A**) and fructose for 5 hours (**B**), furfural from the dehydration of xylose for 6 hours (**C**) at 150 °C.

Under the condition of 150 °C for 4 hours, 5-HMF yield from glucose is in the range of 32.5% to 59.0% with the different Nb_2_O_5_ loading (Fig. 6A), and the best 5-HMF yield is about 59.0% over the catalyst with 0.5 wt% Nb_2_O_5_ loading, while the yield is only 33.5% with 33.9 wt% Nb_2_O_5_ loading. 59.0% 5-HMF yield over 0.5 wt% Nb_2_O_5_-γ-Al_2_O_3_ nanofiber is significant which is higher than those reported in literatures^20^,^25^,^26^. Nb_2_O_5_-γ-Al_2_O_3_ nanofiber can well catalyze the conversion of fructose and xylose to 5-HMF and furfural, which is not as significant as glucose conversion (Fig. 6B,C). As Nb_2_O_5_ loading increased from 3 wt% to 33.9 wt%, 5-HMF yields from fructose conversion raised from 67.4% to 76.8%, but 3 wt% Nb_2_O_5_ loading resulted in the minimum 5-HMF yield. When xylose was dehydrated, the maximum 56.1% furfural yield was obtained over 1 wt% Nb_2_O_5_-γ-Al_2_O_3_ nanofiber, but furfural yield declined to 36.9% when Nb_2_O_5_ loading reached 33.9 wt%. Those results indicated that the niobia species existed on the γ-Al_2_O_3_ nanofiber support played the key role on biomass conversion or dehydration.

**Figure 7:**
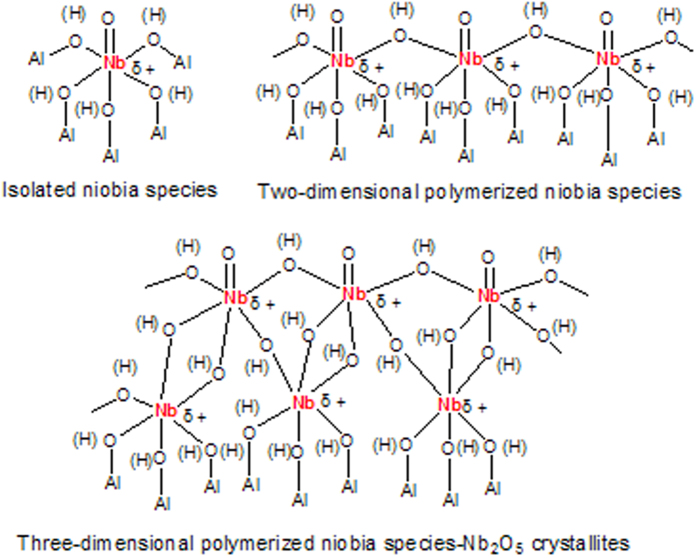
The states of niobia species dispersed on the γ-Al_2_O_3_ nanofibers.

As shown in Fig. 7, the states of niobia species dispersed on the γ-Al_2_O_3_ nanofibers can be expressed in such three kinds of structure as a single NbO_6_ unit, two-dimensional aggregation and three-dimensional aggregation^27^,^28^,^29^,^30^. If the niobia species exist in the form of a highly dispersed monomer NbO_6_ unit, Lewis acid sites are originated from Nb^δ+^ ion. At low Nb_2_O_5_ loading, the niobia species dispersed on the γ-Al_2_O_3_ nanofiber support through Nb–O–Al bridge bonds. γ-Al_2_O_3_ has Lewis acid sites with different acid strengths and weak Brønsted acid sites, and the reaction between Nb_2_O_5_ precursor and hydroxyl groups on the surface of γ-Al_2_O_3_ nanofiber results in strong metal-support interaction, generating Nb_2_O_5_-γ-Al_2_O_3_ nanofiber with both strong Lewis acid sites and relatively intensive Brønsted acid sites^14^. With the increase of Nb_2_O_5_ loading, the interaction between the isolated niobia species and their nearest neighbors (either isolated or polymerized species) resulted in the formation of Nb–O–Nb bridge bonds. The Brönst acid sites originated from the Nb–OH–Nb bridge bonds^27^,^28^,^29^, and the abundance and intensity of Brønsted acid sites could be raised obviously because Nb_2_O_5_ loading increase could lead to the formation of three-dimensional polymerized niobia species. Nb_2_O_5_ crystallization caused a rapid decline of the L and B acid sites of Nb_2_O_5_-γ-Al_2_O_3_ nanofiber, which indicated that the crystalline phase Nb_2_O_5_ has few L and B acid sites^30^.

**Figure 8:**
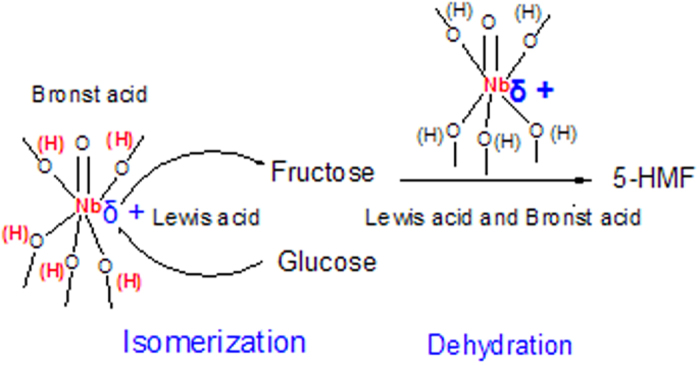
Glucose conversion into 5-HMF over the Nb_2_O_5_-γ-Al_2_O_3_.

It is known that the conversion of glucose to 5-HMF is a two-step reaction. The first step is the isomerization of glucose to fructose catalyzed by Lewis acid and the second step is the dehydration of generated fructose from glucose to 5-HMF under Brønsted acid conditions^31^. Herein, Nb_2_O_5_ loading increase could lead to the formation of two-dimensional polymerized niobia species, three-dimensional polymerized niobia species and crystallization, which influenced the distribution and quantity of the Lewis acid sites and Brønsted acid sites. On one hand, the Lewis acid site Nb^δ+^ play a key role on the isomerization of glucose to fructose, and Brønsted acid sites are more active in the dehydration of generated fructose to 5-HMF^14^,^32^. The heterogeneous catalyst with the suitable ratio of Lewis acid sites to Brønsted sites should display an more excellent catalytic performance in the conversion of glucose to 5-HMF in organic solvents^33^. Herein, the γ-Al_2_O_3_ nanofibers loaded with 0.5~1 wt% Nb_2_O_5_ offers the optimum ratio of Lewis acid sites to Brønsted acid sites, thus they exhibits the best performance in 5-HMF (or furfural) yield from glucose (or xylose) (see Fig. 8). On the other hand, the 1D γ-Al_2_O_3_ nanofiber support may play an important role on improving 5-HMF yield. For instance, the active Nb_2_O_5_ catalytic centers are decorated on the external surface of γ-Al_2_O_3_ fibers, improving the direct interaction between the active sites and glucose. The randomly oriented nanofibers form a large interconnected void (10~20 nm), which made glucose to well contact with the active sites^34^.

**Figure 9:**
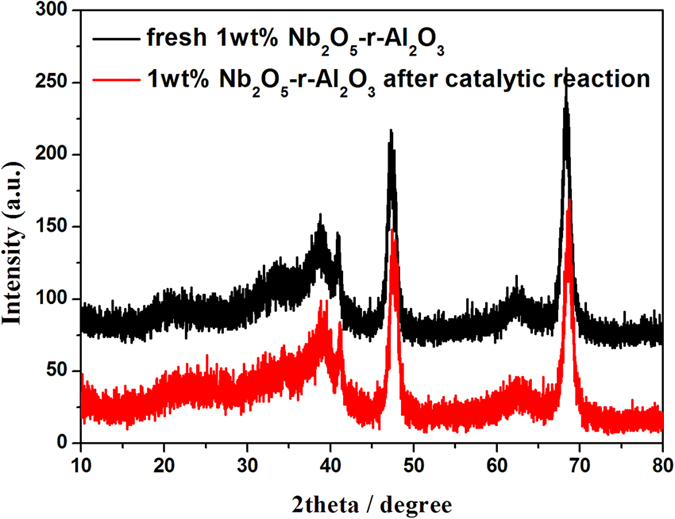
XRD of 1 wt% Nb_2_O_5_-γ-Al_2_O_3_ before and after the catalytic reaction.

**Figure 10:**
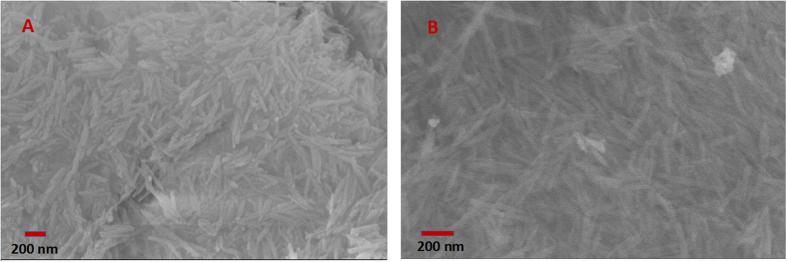
SEM of 1 wt% Nb_2_O_5_-γ-Al_2_O_3_ before (**A**) and after the catalytic reaction (**B**).

The catalyst re-usability was studied using 1 wt% Nb_2_O_5_-γ-Al_2_O_3_ nanofibers. After reaction, the catalyst was separated from DMSO by centrifugation, and then washed with deionized water and ethanol, dried at 80 °C under vacuum before the next run. From Figs 9 and 10, it is found that the XRD pattern and morphology of catalyst well maintain after one recycle. However, the color of catalyst changed from white to brown, which maybe result from an accumulation of humans on the surface of catalyst^12^, which caused some decrease of catalytic performance.

## Conclusions

Nb_2_O_5_-γ-Al_2_O_3_ nanofibers have been prepared by facile incipient-wetness impregnation method to catalyze the conversion of glucose (fructose and xylose as well) into 5-HMF. It is found that Nb_2_O_5_/γ-Al_2_O_3_ nanofibers can efficiently promote the dehydration of glucose, fructose and xylose. The sample with 0.5~1 wt% Nb_2_O_5_ load exhibits the best performance in glucose conversion into 5-HMF, and 5-HMF yield come up to 55.9~55.9%. This excellent performance of 0.5~1 wt% Nb_2_O_5_/γ-Al_2_O_3_ nanofibers in glucose conversion into 5-HMF is ascribed to the synergistic effect of suitable ratio of Lewis acid sites to Brønsted acid sites on Nb_2_O_5-_γ-Al_2_O_3_ nanofibers.

## Methods

### Synthesis of supports

All commercially available chemicals and solvents are of reagent grade and were used as received without further purification. The γ-Al_2_O_3_ nanofibers were prepared by the hydrothermal method. A buffer solution prepared by diluting ammonia (40 mL, 25%) with deionized water to 10%, was used as the precipitation agent. Besides, 30 g Al(NO_3_)_3_·9H_2_O was dissolved in 50 mL deionized water. The buffer solution was loaded into the solution of Al(NO_3_)_3_ by dropwise under vigorous stirring until the solution became milky and the initial pH of the mixture ranged from 2.0 to 5.0. The resulting uniform solution was then transferred into a PTFE-lined autoclave and heated in an oven at 200 °C for 48 h. Thereafter, the obtained precipitate was washed several times with deionized water and ethanol by centrifugation, and the obtained precipitate was dried overnight at 55 °C and subsequently calcined in air at 600 °C for 5 h to obtain γ-Al_2_O_3_ nanofibers.

### Preparation of catalysts

Nb_2_O_5_-γ-Al_2_O_3_ nanofibers were prepared by the incipient-wetness impregnation method where NbCl_5_ was selected as the niobium precursor and incorporated into γ-Al_2_O_3_ nanofibers. Firstly, the appropriate amount of NbCl_5_ was mixed together with the prepared γ-Al_2_O_3_ nanofibers (0.5 g) in order to obtain catalysts with the controlled Nb_2_O_5_ loading [wt% = Nb_2_O_5_/(Nb_2_O_5_ + Al_2_O_3_)] equal to 1, 3, 5, 10, 15, 30 and 40, respectively. Secondly, the deionized water containing the oxalate with the mole about five times of the mole of NbCl_5_ was introduced and then the mixture was kept at room temperature for 48 h. Thirdly, the mixture was dried at 100 °C for 24 h to obtain the different Nb_2_O_5_-γ-Al_2_O_3_ catalysts.

### Catalytic activity

The glucose, fructose and xylose dehydration reactions were performed in a 15 mL sealed tube (thick walled pressure bottle from Beijing synthware glass) under magnetic stirring. In a typical run, glucose (450 mg), catalyst (45 mg) and DMSO (2.5 ml) were loaded into sealed tube which was then immersed into the preheated oil bath and stirred for a required time. After reaction, the mixture cooled to room temperature naturally, and then the internal standard substances (1-chloronaphthalene) was added into reaction mixture which was further diluted by methanol. The filtered solution was analyzed by HPLC. The dehydration reaction procedures of fructose and xylose were similar to that of glucose, and the glucose (450 mg) was replaced by fructose (450 mg) or xylose (375 mg), respectively.

### General Information

The surface morphology and composition of catalysts were characterized by field emission scanning electron microscopy (SEM, JSM-7001F, JEOL, Tokyo, Janpan). High-resolution transmission electron microscopy (HRTEM) images were taken on a JEOL JEM-2100F field emission electron microscope under an accelerating voltage of 200 kV equipped with an energy-dispersive X-ray spectroscopy (EDX) instrument (Quantax-STEM, Bruker). The phases structures of catalysts were characterized by powder X-ray diffraction (XRD) analysis using an X-ray diffractometer (DX-2700, China) with Ni-filtered Cu Kα radiation (λ = 1.5406 Å) at 40 kV and 30 mA with a fixed slit, ranging from 10 to 80°. Surface areas were determined by low temperature N_2_ adsorption performed at 77 K, on a 3H-2000PS2 analysis instrument, after pretreatment performed for 8 h at 150 °C under vacuum. The BET (Brunauere-Emmete-Teller) method was used to derive surface areas from the resulting isotherms. Pore size distributions were obtained from analysis of the adsorption branch of the isotherms using Barrette Joynere Halenda (BJH) method. The Raman spectra of these catalysts were determined by Renishaw inVia plus from 200 to 2000 cm^−1^. The Nb_2_O_5_ contents of Nb_2_O_5_-γ-Al_2_O_3_ nanofibers were characterized by Optima 8000 (ICP-AES). The 5-HMF and furfural were determined by high performance liquid chromatography (HPLC) (L6, China) fitted with a Pgrandsil-TC-C18 column and the ultraviolet detectors for 5-HMF and furfural at 286 nm and 272 nm, respectively. The column oven temperature was set at 25 °C, and the mobile phase was methanol/water = 80:20 (V/V) at a flow rate of 1.0 mL min^−1^.

## Additional Information

**How to cite this article**: Jiao, H. *et al.* Nb_2_O_5_-γ-Al_2_O_3_ nanofibers as heterogeneous catalysts for efficient conversion of glucose to 5-hydroxymethylfurfural. *Sci. Rep.* **6**, 34068; doi: 10.1038/srep34068 (2016).

## Figures and Tables

**Table 1:** The Nb_2_O_5_ contents of Nb_2_O_5_-γ-Al_2_O_3_ by ICP-AES.

Catalyst	Nb^5+^ concentration (mg/L)	Nb_2_O_5_ content controlled (wt%)	Nb_2_O_5_ real content (wt%)
1 wt%Nb_2_O_5_-Al_2_O_3_	0.046	1	1.0
3 wt%Nb_2_O_5_-Al_2_O_3_	0.014	3	3.0
5 wt%Nb_2_O_5_-Al_2_O_3_	0.016	5	3.4
10 wt%Nb_2_O_5_-Al_2_O_3_	0.022	10	4.7
15 wt%Nb_2_O_5_-Al_2_O_3_	0.044	15	9.4
30 wt%Nb_2_O_5_-Al_2_O_3_	0.124	30	26.6
40 wt%Nb_2_O_5_-Al_2_O_3_	0.158	40	33.9

**Table 2:** The textural properties of γ-Al_2_O_3_ with different Nb_2_O_5_ loadings.

Sample (wt%)	BET surface (m^2^ g^−1^)	Pore volume (cm^3^ g^−1^)	Average pore diameter (nm)	
			BJH adsorption	BJH desorption
0	70.86	0.63	26.93	26.42
1	74.02	0.61	27.79	25.39
3	73.36	0.55	25.30	24.92
3.4	81.75	0.48	22.86	23.70
4.7	116.49	0.46	17.21	21.88
9.4	130.99	0.32	10.77	14.29
26.6	123.18	0.25	1.45	1.85
33.9	103.63	0.23	1.40	1.90
